# Supplementary material for: Systematic Investigation of Aluminum Stress-Related Genes and Their Critical Roles in Plants
Source: Int J Mol Sci. 2024 Aug 21;25(16):9045. doi: 10.3390/ijms25169045 (PMC11354972; doi:10.3390/ijms25169045)
Supplement: Supplementary file 1 [file ijms-25-09045-s001.zip › ijms-3153797-supplementary.pdf]

Supplementary Materials

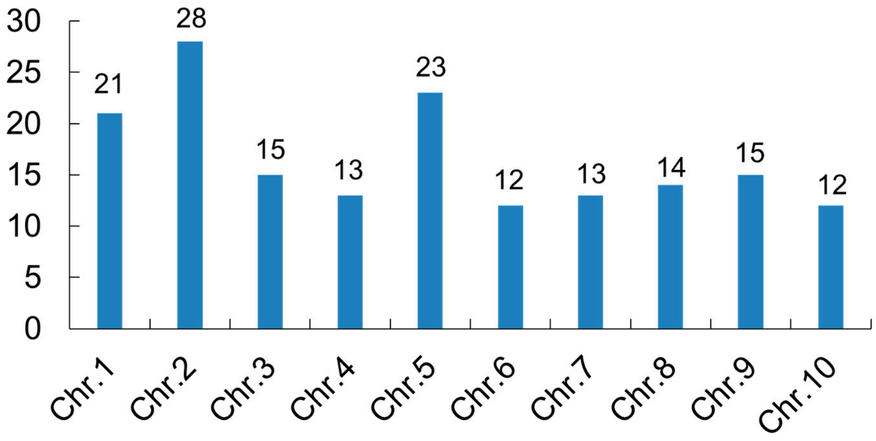

Figure S1. The number of the predicted maize aluminum stress-related genes located in the 10 chromosome of maize.

Table S1. Analysis of the characteristic and orthologues in maize for the reported Al-SR genes in *Arabidopsis*, rice, wheat, maize, and sorghum.

No.	Gene Names	Orthologues in Maize	Expression Organ	Subcellular localization	Aluminum tolerance	References
**Transporters**						
1	AtSTAR1	Zm00001eb226430	Roots and shoots	Vesicle membrane	+	[31]
2	OsSTAR1	Zm00001eb226430	Roots	Vesicle membrane	+	[32]
3	SbSTAR1	Zm00001eb226430	Roots	Cytoplasm and nucleus	+	[20,21]
4	OsSTAR2	-	Roots	Vesicle membrane	+	[32]
6	AtALS1	Zm00001eb214670	Roots	Vacuolar membrane	+	[33]
7	OsALS1	Zm00001eb214670	Roots	Tonoplast	+	[34]
5	AtALS3	-	Roots, cotyledons, leaves, stems, and flowers	Plasma membrane	+	[78]
8	ZmPGP1	-	Roots	Plasma membrane	-	[35]
9	AtMATE	-	Mature portions of the roots	Plasma membrane	+	[36,37]
10	SbMATE	-	Roots	Plasma membrane	+	[20,43-47,49-51]
11	ZmMATE1	-	Roots	Plasma membrane	+	[52,53,79]
12	AtFRDL3	Zm00001eb008790, Zm00001eb143800, Zm00001eb261140, Zm00001eb424530	Roots	Plasma membrane	+	[38,39]
13	OsFRDL2	Zm00001eb143800, Zm00001eb261140, Zm00001eb424530	Roots	Vesicle	+	[40]
14	TaMATE2	Zm00001eb143800, Zm00001eb261140, Zm00001eb424530	Roots	Plasma membrane	+	[54]
15	ZmMATE2	-	Roots	Plasma membrane	+	[79]
16	ZmMATE6	-	Roots, and leaves	Cytoplasm and the nucleus	+	[22]
17	OsFRDL4	-	Roots	Plasma membrane	+	[41,42,80]

18	AtALMT1	Zm00001eb083510, Zm00001eb422120	Roots	Plasma membrane	+	[36·37·55-58]
19	TaALMT1	Zm00001eb083510, Zm00001eb422120	Roots	Plasma membrane	+	[57·62·65·81-83]
20	OsALMT4	-	Roots, shoots, leaves, anthers,stigma	Plasma membrane	+	[63·64]
21	AtALMT9	-	-	Vacuolar channel	+	[60·61]
22	AtALMT12	Zm00001eb274380	Leaves, roots	Endomembranes and plasma membrane	+	[62·63]
23	OsNrat1	Zm00001eb231210	Roots	Plasma membrane	+	[66-68]
24	SbNrat1	Zm00001eb231210	Roots, shoots	Plasma membrane	+	[69]
25	ZmNRAMP4	-	Roots	Plasma membrane	+	[70]
26	OsMGT1	-	Roots, Shoots	Cytoplasm and nucleus	+	[23]
27	OsPIN2	Zm00001eb389260	Roots	Plasma membrane	+	[71·72]
31	OsAUX3	Zm00001eb288800	Roots	Plasma membrane	-	[73]
28	AtOT	Zm00001eb054370	Roots, stems, and leaves	Plasma membrane	+	[74]
29	AtNIP1;2	Zm00001eb239170, Zm00001eb384790, Zm00001eb384800	Roots	Plasma membrane	+	[75·84]
30	OsNIP1;2	-	Seed, root, stem, leaf, and panicle	Plasma membrane	+	[77]
**Transcription factors**						
1	OsART1	Zm00001eb408000	Roots	Nucleus	+	[9·10·15-17]
2	OsART2	Zm00001eb038910	Roots	Nucleus	+	[9]
3	AtSTOP1	Zm00001eb146420, Zm00001eb366580, Zm00001eb408000	Roots	Nucleus	+	[11·12·14·85]
4	SbSTOP1a	Zm00001eb063930	Roots	Nucleus	+	[86]
5	SbSTOP1b	Zm00001eb244720	Roots	Nucleus	+	[86]
6	SbSTOP1c	Zm00001eb038910	Roots	Nucleus	+	[86]
7	SbSTOP1d	Zm00001eb366580	Roots	Nucleus	+	[86]
8	TaSTOP1	Zm00001eb146420, Zm00001eb366580	-	Nucleus	+	[87]
9	AtSTOP2	-	Roots	Nucleus	+	[88]
10	SbZNF1	Zm00001eb289180	Tips, roots, shoots	Nucleus	+	[48]
11	SbWRKY1	Zm00001eb352050	Tips, roots	Nucleus	+	[48]
12	OsWRKY22	Zm00001eb149600	Roots	Nucleus	+	[42]
13	AtWRKY46	Zm00001eb042020, Zm00001eb098330, Zm00001eb158000, Zm00001eb178550, Zm00001eb310260, Zm00001eb310270, Zm00001eb344160, Zm00001eb359470	Roots	Nucleus	-	[89]
14	AtWRKY47	Zm00001eb129690, Zm00001eb332300, Zm00001eb417490	Roots	Nucleus	+	[90]
15	SbWRKY22	Zm00001eb330710	Roots	Nucleus	+	[20]
16	SbWRKY65	Zm00001eb154170	Roots	Nucleus	+	[48]
17	OsASR1	Zm00001eb131490, Zm00001eb243310	Roots	Nucleus and cytoplasm	+	[24]
18	OsASR5	-	Roots	Nucleus and cytoplasm	+	[24·91·92]
19	AtHB7	Zm00001eb075230, Zm00001eb103330, Zm00001eb248930, Zm00001eb319390,	Roots	Nucleus	-	[93]

		Zm00001eb427650				
20	AtHB12	Zm00001eb075230, Zm00001eb103330, Zm00001eb248930, Zm00001eb319390, Zm00001eb427650	Roots	Nucleus	-	[93]
21	SbHY5	Zm00001eb235510	Roots	Nucleus	+	[94]
22	OsMYB30	Zm00001eb099830, Zm00001eb313580	Roots	Nucleus	-	[95]
23	AtMYB103	Zm00001eb416400	Roots	Nucleus	+	[96]
24	AtNAC017	-	Roots, stems, leaves, flowers, and siliques	Nucleus	-	[97]
25	AtSOG1	Zm00001eb308090	Roots	Nucleus	+	[98,99]
26	AtMYC2	-	Roots	Nucleus	+	[100]
27	AtLUH	Zm00001eb065280, Zm00001eb258660	Roots	Nucleus	-	[101,102]
28	AtSLK2	-	Roots	Nucleus	-	[101]
29	AtPIF4	Zm00001eb059460, Zm00001eb070520, Zm00001eb334310, Zm00001eb431080	Roots	Nucleus	-	[7]
30	AtRBR1	Zm00001eb037120, Zm00001eb113470, Zm00001eb197990, Zm00001eb198220	Roots	Nucleus	+	[103]
**Kinases/phosphatase**						
1	AtWAK1	Zm00001eb071980	Roots	Plasma mem-brane	+	[104]
2	AtCK2	Zm00001eb023910, Zm00001eb105560, Zm00001eb322050, Zm00001eb390860	Roots	Chloroplast	+	[105]
3	AtRAE1	Zm00001eb094050, Zm00001eb163310	Roots, rosette leaves, cauline leaves, flower tis-sues, and siliques	Nucleus	+	[6,106]
6	AtRAH1	-	Roots, cotyledons, rosette leaves and cauline leaves, flower tissues and siliques	Nucleus	-	[106]
4	AtRAE2	Zm00001eb309300	Roots, leaves	Nucleus	+	[107]
5	At-RAE3/AtHPR1	Zm00001eb000780	Roots, old and young leaves, stems, flowers, and siliques	Nucleus	+	[107,108]
7	AtESD4/RAE5	Zm00001eb016540, Zm00001eb111330, Zm00001eb131540, Zm00001eb341180	Roots	Nucleus	-	[109]
8	AtSIZ1	Zm00001eb163160, Zm00001eb265410, Zm00001eb355110	Roots	Nucleus	-	[106,109-111]
9	AtMEKK1	-	-	Plasma mem-brane	+	[112]
10	AtMKK1	Zm00001eb377050	Leaves	-	+	[112]
11	AtMKK2	Zm00001eb377050	Leaves	-	+	[112]
12	AtMPK4	Zm00001eb393610, Zm00001eb415940	Phloems, leaves, edges and sto-mata	-	-	[112]
13	OsSAL1	Zm00001eb228190, Zm00001eb272240	Roots, leaves, stems, and spikes	Plasma mem-brane	+	[27]

14	AtPP2C.D5	Zm00001eb003000, Zm00001eb003010, Zm00001eb003020, Zm00001eb228190, Zm00001eb272240, Zm00001eb393920, Zm00001eb402720	Shoots, roots, leaves	Nucleusi and the cytosol	-	[27,28]
15	AtPP2C.D6	Zm00001eb003000, Zm00001eb003010, Zm00001eb003020, Zm00001eb228190, Zm00001eb272240, Zm00001eb393920, Zm00001eb402720	Shoots, roots, leaves	Nucleusi and the cytosol	-	[27,28]
16	AtPP2C.D7	Zm00001eb003000, Zm00001eb003010, Zm00001eb003020, Zm00001eb228190, Zm00001eb272240, Zm00001eb393920, Zm00001eb402720	Shoots, roots, leaves	Nucleusi and the cytosol	-	[27,28]
17	OsA7	Zm00001eb049410, Zm00001eb049470, Zm00001eb049510, Zm00001eb067750, Zm00001eb307890, Zm00001eb432500	Roots	Plasma membrane	+	[27]
18	AtPAH1	Zm00001eb289800	-	Endoplasmic reticulum	-	[113]
19	AtPAH2	Zm00001eb166810	-	Endoplasmic reticulum	-	[113]
20	AtATR	Zm00001eb228310	Roots	Nucleus	+	[114]
21	OsArPK	-	Roots, shoots	Plasma membrane	+	[115]
**Sugar metabolism**						
1	OsEXPA10	Zm00001eb072890, Zm00001eb428030	Roots and shoots	-	-	[116]
2	ZmXTH	-	Roots	Cell wall	+	[117]
3	AtXTH15	Zm00001eb226450, Zm00001eb226490, Zm00001eb341630	Roots	Cell wall	-	[118]
4	AtXTH31	Zm00001eb000660, Zm00001eb022710	Roots	Plasma membrane	-	[119]
5	AtTBL27	Zm00001eb164580, Zm00001eb279870, Zm00001eb279880, Zm00001eb371970	Leaves, stems, flowers, and siliques	Golgi	+	[96,120]
6	AtPME46	Zm00001eb256250	Roots	-	+	[101]
7	AtPARVUS	-	-	Endoplasmic reticulum	-	[121]
8	SbGLU1	Zm00001eb329710	Roots	Cytoplasm and Nucleus	+	[20,26,122]
**Hormone-related**						
1	AtEIN2	Zm00001eb054060, Zm00001eb119690, Zm00001eb216860	Roots	Nucleus, cytoplasm, and endoplasmic reticulum	-	[19]
2	AtYUC9	Zm00001eb098150	Roots	-	-	[123]
3	AtYUC8	-	Roots	-	-	[123]
4	AtYUC7	-	Roots	-	-	[123]
5	AtYUC3	-	Roots	-	-	[123]
6	AtYUC5	Zm00001eb098150	Roots	-	-	[123]
7	AtTAA1	Zm00001eb123040, Zm00001eb336530	Roots	Cytoplasm	-	[7,124]
8	AtCOI1	Zm00001eb011780, Zm00001eb090100, Zm00001eb147720, Zm00001eb288770, Zm00001eb347860, Zm00001eb367310,	Roots	Nucleus	-	[100]

		Zm00001eb397990				
9	AtSUR1	Zm00001eb113920, Zm00001eb201770, Zm00001eb203230, Zm00001eb240650, Zm00001eb385390	Roots	-	+	[118,125]
10	AtSUR2	Zm00001eb164760, Zm00001eb408580	Roots	-	+	[118,126]
11	AtNPR1	Zm00001eb121040	Roots, and leaves	Cytoplasm and nucleus	+	[19]
**ROS metabolism**						
1	OsApx1	Zm00001eb028900, Zm00001eb102190, Zm00001eb190040, Zm00001eb210050, Zm00001eb294460, Zm00001eb315550, Zm00001eb367350, Zm00001eb413440	Roots	Cytoplasm	-	[127]
2	OsApx2	Zm00001eb112040	Roots	Cytoplasm	-	[127]
3	AtGR1	Zm00001eb258600	Roots	Cytoplasm	+	[128]
4	AtGST1	-	Leaves, shoots	Cytoplasm	+	[129]
5	AtGST11	-	Leaves, shoots	Cytoplasm	+	[129]
6	AtPrx64	Zm00001eb240670	Roots	Cytoplasm	+	[130]
7	AtAOX1a	Zm00001eb071870, Zm00001eb071880, Zm00001eb071890, Zm00001eb251680	Roots	Mitochondria	+	[131]
8	ZmAT6	-	Roots, shoots	Chloroplast	+	[132]
9	ZmALDH	-	Roots, leaves	Plasma membrane, nucleus	+	[25]
10	AtNADP-ME1	-	Roots, leaves	Cytoplasm	-	[133]
**Other processes**						
1	AtGRP3	-	Roots	-	-	[134]
2	AtCBL1	Zm00001eb023890, Zm00001eb390900	Roots, shoots	Plasma membrane	+	[135]
3	AtALS7	Zm00001eb032180	Roots	Nucleus	+	[136]
4	AtSWA2	Zm00001eb008900	Roots	Nucleus	+	[136]
5	OsGERLP	Zm00001eb005320, Zm00001eb402400	Roots, stems, and leaves	Nucleus	+	[137]
6	AtVHA-a2	Zm00001eb010790, Zm00001eb398530	Roots	Vacuole membrane	-	[8]
7	AtVHA-a3	Zm00001eb010790, Zm00001eb398530	Roots	Vacuole membrane	-	[8]
8	AtRAD51	Zm00001eb163340, Zm00001eb234890, Zm00001eb237850, Zm00001eb267780	Roots	Nucleus	+	[103]
9	AtCYCB1	Zm00001eb168940, Zm00001eb305730, Zm00001eb309490, Zm00001eb317280, Zm00001eb332550	Roots	Nucleus	+	[103]
10	AtSUV2	-	Roots	Nucleus	-	[114]
11	At-TANMEI/ALT2	-	Roots	Nucleus	+	[138]
12	AtPGIP1	-	Shoots, roots, leaves	-	+	[139]
13	AtALT1	Zm00001eb074540, Zm00001eb074550	Roots, leaves and seeds	-	-	[78]
14	OsRAL1/4CL4	Zm00001eb389420	Stems, Leaves	Cytoplasm	+	[140,141]
15	Os4CL3	Zm00001eb233720	Stems, roots, leaves, and flowers	Cytoplasm	+	[140,142]
16	Os4CL5	Zm00001eb040790	Stems, roots, leaves, and flowers	Cytoplasm	+	[142]

17	OsCS1	Zm00001eb206090, Zm00001eb206100, Zm00001eb236450	-	mitochondria	+	[143]
18	TaWali1	-	Roots, Shoots	Nucleus	+	[144]
19	TaWali5	-	Roots, Shoots	Nucleus	+	[144]

Note: The orthologues in maize are based on ensemble plants website (https://plants.ensembl.org/index.html. (accessed on 26 February 2024))
